# Can the size of the contact area body-ground influence the presence of acute pulmonary emphysema in cases of incomplete hanging?

**DOI:** 10.1007/s00414-023-02998-z

**Published:** 2023-04-26

**Authors:** C. Castiglioni, F. Lanzillotta, T. Fracasso

**Affiliations:** 1 Unit of Forensic Medicine, University Center of Legal Medicine Lausanne-Geneva, Lausanne University Hospital and University of Lausanne, Chemin de La Vulliette 4, 1000 Lausanne, Switzerland; 2 Local Health Public Utility – “U. Parini Hospital”, Via Ginevra 3, 11100 Aosta, Italy; 3 Unit of Forensic Medicine, University Center of Legal Medicine Lausanne-Geneva, Geneva University Hospital and University of Geneva, Rue Michel-Servet 1, 1211 Geneva, Switzerland

**Keywords:** Forensic histopathology, Acute pulmonary emphysema, Incomplete hanging, Sign of vitality, Morphometric digital analysis

## Abstract

In a recently published study, we showed that acute pulmonary emphysema (APE) was present in cases of death by incomplete hanging and absent in cases of complete hanging. This result suggested a possible role of the hanging position on the respiratory distress of these victims. To further investigate this hypothesis, in the present study, we compared cases of incomplete hanging with a small contact area between body and ground (group A) to cases of incomplete hanging with a large contact area (group B). As positive and negative control group, we investigated cases of freshwater drowning (group C) and acute external bleeding (group D) respectively. Pulmonary samples were histologically examined, and the mean alveolar area (MAA) for each group was measured by digital morphometric analysis. MAA was 23,485 μm^2^ for group A and 31,426 μm^2^ for group B (*p* < 0.05). MAA of group B was similar to MAA of positive control group (33,135 μm^2^) and MAA of group A was similar to MAA of negative control group (21,991 μm^2^). These results seem to confirm our hypothesis and suggest that the size of the contact area between body and ground influences the presence of APE. Furthermore, the present study showed that APE can be proposed as a vitality sign in incomplete hanging, but only in cases with a large contact area between body and ground.

## Introduction

In death by mechanical airway obstruction, such as manual or ligature strangulation, suffocation, inhalation of blood or gastric content and drowning, repeated and violent inspiration attempts lead to an increase of the negative intra-thoracic pressure with subsequent acute pulmonary emphysema (APE). APE is considered as a sign of vitality in numerous cases of mechanical asphyxia and is frequently reported in the literature [1–5].

In death by hanging airway, compression seems to play a minor role if compared to cerebral hypoperfusion due to cervical vascular compression [6, 7]. In cases of hanging, APE is rarely reported in the literature based on macroscopic examination of lung or on conventional histological examination of lung tissue [8]. In a previous study, we showed the presence of APE in cases of incomplete hanging, by morphometric digital analysis of lung tissue [9]. This result suggested a possible role of the body position in influencing the occurrence of APE in death by hanging.

To further investigate this hypothesis, in the present study, we focused our attention on cases of incomplete hanging. Indeed this is a heterogeneous group including cases in which the body is almost completely suspended, only the tips of the toes touching the ground, and cases in which the body is almost lying on the ground, with a much larger area of the body in contact with the ground. We hypothesize that the size of the contact area between body and ground has an influence on the pulmonary distress and as consequence on the presence or absence of APE.

## Materials and methods

We selected cases of incomplete hanging with a small contact area between body and ground (group A: contact area equal to or less than the soles of the both feet) and cases of incomplete hanging with a large contact area (group B: contact area larger than the soles of the both feet). Cases of freshwater drowning were investigated as positive control group (group C) and deaths by acute external bleeding as negative control group (group D). All cases have been retrospectively selected from the autopsy records of the University Center of Legal Medicine of Geneva (Switzerland). For every case of hanging included in this study, the body position was photographically documented by the forensic pathologist called by the police for scene investigation.

To limit the impact of confounders, several exclusion criteria have been applied: age greater than 65 years, inhalation of blood and/or gastric content, chronic respiratory cardiac diseases (according to clinical and autopsy records), cardiopulmonary resuscitation, signs of putrefaction, and postmortem interval (PMI) longer than 72 h. Chronic pulmonary emphysema was excluded by collecting information from the clinical records, post-mortem imaging investigations, macroscopic, and microscopic lung tissue examination.

One peripheral sample of pulmonary tissue from the upper and the lower lobes, in non-hypostatic areas, was collected and fixed in 4% saline-buffered formalin solution for 24 h, then dehydrated through alcohol and paraffin-embedded. Histological slides were stained with hematoxylin–eosin (HE) and examined by an optical microscopy (Nikon Eclipse 50i). According to our previous study, we used the area of alveoli and alveolar ducts as the best morphological parameter to investigate the APE.

Before morphometric analysis, histological slides were anonymized to avoid any bias in the field selection and measurements.

### Morphometric digital analysis

For each slide, we randomly selected 5 fields (magnification 10 ×). Each field was photographed by high-resolution microscope camera (Nikon Digital Sight DS-Fil). Captured images were analyzed, and the area of all alveoli and alveolar ducts was measured by image analysis software (Nikon Elements BR 3.2). As showed in our previous study, this software can easily detect the alveolar walls and measure the alveolar area with high accuracy even with alveolar edema or intra-alveolar cells. All detectable alveolar spaces in each picture were measured excluding the edges of the field. To avoid artifacts such as air bubbles in blood vessels, the alveolar spaces were manually selected one by one. The area was automatically calculated and exported to MS Excel Table. The mean alveolar area (MAA) was calculated for each case and for each group. A *T* test used to compare the MAA between the groups. Statistical significance was fixed at *p* values < 0.05.

## Results

We included 10 cases for the group A, 10 cases for the group B, 10 cases for the group C, and 10 cases for the group D. Demographic data of the groups are shown in Table 1.

**Table 1:** The demographic data

	No	Sex	Mean age	Mean PMI
Group A	10	6 male 4 female	41y (range 21–61)	40 h
Group B	10	8 male 3 female	43y (range 30–56)	39 h
Group C	10	6 male 4 female	36y (range 27–53)	38 h
Group D	10	8 male 2 female	41y (range 38–60)	40 h

**Fig. 1:**
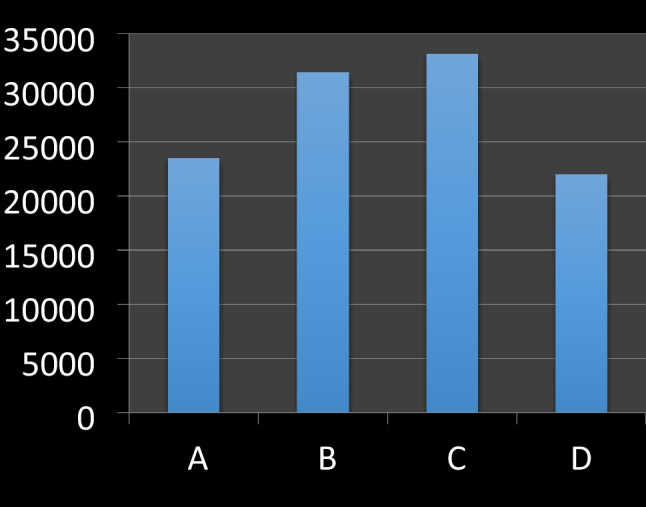
Mean alveolar area of each group: between the groups A and B of incomplete hanging there is a statistically significant difference (*p* < 0.05)

We investigated 160 slides and 800 fields and measured a total of more than 25,000 alveolar spaces. The number of measurements performed for each group and the values of MAA for each case and each group are reported in Table 2 and Fig. 1.

**Table 2:** Mean alveolar area of each case

Number of measurements	A 6933	B 5455	C 5947	D 7457
MAA for each case (μm^2^)				
Case no. 1	12,166	26,269	51,648	18,049
Case no. 2	32,436	35,940	24,909	24,705
Case no. 3	24,444	30,494	28,404	22,842
Case no. 4	27,063	38,954	27,260	16,711
Case no. 5	17,370	34,392	30,611	16,144
Case no. 6	22,199	20,529	30,748	30,379
Case no. 7	23,497	24,992	31,937	18,955
Case no. 8	27,904	33,906	39,885	28,457
Case no. 9	24,717	37,462	34,841	20,570
Case no. 10	22,789	31,322	31,108	23,106

MAA was 23,485 μm^2^ for group A and 31,426 μm^2^ for group B. Between these two groups of incomplete hanging, there is a statistically significant difference (*p* < 0.05).

MAA of group B was similar to MAA of group C (33,135 μm^2^). Furthermore, MAA of group A was similar to MAA of group D (21,991 μm^2^).

## Discussion

The present study showed a very important difference in incomplete hanging between cases with a small contact area between body and ground and cases with a large contact area. In the group of incomplete hanging with a large contact area, the APE was very important, similar to cases of freshwater drowning. Instead, in cases of incomplete hanging with a small contact area the APE was absent, as we have observed in our previous study in death by complete hanging [9].

These results seem to confirm our hypothesis that the presence of APE in hanging depends on the position of the body, in particular on the size of the contact area between body and ground. To the best of our knowledge, this is the first time such an observation is reported.

In a study published in 2001, Khoklov calculated the tension exerted on the neck by the ligature in different positions of incomplete hanging by a mathematical model [10]. Khoklov showed that in standing incomplete hanging, in which the body touches the ground only with the toes, the ligature tension is maximal (98.3% of the body weight). The ligature tension gradually decreases in others positions. In kneeling position, the ligature tension is equal to 65.5% of the body weight and in lying position equal to 18.3%.

A low ligature tension logically leads to a minor compression of the cervical vessel, especially of the arteries, possibly resulting in a longer period of agony and as consequence in a major respiratory distress. This could explain why we detected APE in cases where, according to the study of Khoklov, the ligature tension is minor.

Another possible explanation for our results is that a high ligature tension could lead to a major stress on the phrenic nerve roots that start from the cervical spine, with a possible diaphragmatic paralysis. Davies made this hypothesis in a study published in 2010 [11]. In our study, a diaphragmatic paralysis could explain why the APE is absent in cases where the ligature tension is high.

The results of our studies strengthen the idea that the classification of hanging (complete and incomplete) has important pathophysiological implications. Furthermore, incomplete hanging seems to be an inhomogeneous group of death in which circulating and breathing component can be very variable.

Our study confirms that the APE can be interpreted as a sign of vitality in cases of incomplete hanging with large contact area between body and ground.

However, we stress the accent on the high number of potential confounders that we had to take into account as exclusion criteria, which can hinder the use of APE as a vital sign in the routine forensic practice. A future study including a higher number of cases and post-mortem radiological investigations with measurement of the lung volume would be useful to confirm the results of our research.
